# Renal Lesions Following Cerebal Hemorrhage

**Published:** 1874-07

**Authors:** 


### Renal Leisons following Cerebral Hæmorrhages.

Dr. A. Olliver (*Archiv Generales de Med.*, Feb., 1874,) publishes a series of clinical observations and physiological experiments in regard to the dependence of renal congestions and apoplexies, on cerebral hæmorrhages. In his experiments, which were made on rabbits, he endeavored to reproduce the conditions which he found in his observations of his human patients; they are therefore the complement to the latter. He was able, by lacerating the cerebral substance in one hemisphere, to produce congestion and albuminuria in the kidney of the corresponding side; and by causing a meningeal hæmorrhage of the superior longitudinal sinus, to establish a bilateral congestion with albuminuria. By making a puncture in the latteral half of the floor of the fourth ventricle, either an unilateral congestion was produced or a bilateral one, but most pronounced in the kidney of the side corresponding to the cerebral lesion.

Dr. Oliver rapidly reviews his clinical observations as follows:

"The hæmorrhage with only a single exception, had always for its point of departure the left hemisphere, and generally the parts adjoining the fissure of Sylvius. It was constantly accompanied by a slight subarachnoid sanguine effusion over the surface of the neighboring convolutions.

"In all the cases the corpus opto-striata was either destroyed or almost separated from the encephalon by the clot. The clot was always of considerable size, its anterior border either extended to the peduncles or the superior part of the protuberance, or with a circumscribed clot in the corpus opto-striata there existed disseminated *foyers* in the peduncles, or the protuberance. There was found, moreover, in all cases, either a laceration of the septum lucidum, a distention or laceration of the ventricular walls, or the aqueduct of Sylvius was found filled with blood.

"It will be observed therefore, from these facts, that the albuminuria may follow not only hæmorrhage in the protuberance, but also that in other parts of the encephalon.

"Albuminuria of cerebral origin is much more frequent than is at present believed; and I do not doubt that new reaserches will reveal many examples. In the present actual state of our knowledge it is not yet possible to fix with precision, from the symptoms, the location of a cerebral hæmorrhage. Nevertheless, in the cases where the signs of a lesion of the protuberance are wanting, we may say that the presence of albumen in the urine indicates, perhaps, a *foyer* situated at the base of the brain, or perhaps an extensive hæmorrhage compressing the base. In all cases it seems to be, from the facts I relate, a prognostic sign of very grave significance."—*Chicago Journal Nervous Disease.*

